# Quantification of diaphragmatic dynamic dysfunction in septic patients by bedside ultrasound

**DOI:** 10.1038/s41598-022-21702-6

**Published:** 2022-10-15

**Authors:** Yunqiu Chen, Yujia Liu, Mingxin Han, Shuai Zhao, Ya Tan, Liying Hao, Wenjuan Liu, Wenyan Zhang, Wei Song, Mengmeng Pan, Guangyu Jiao

**Affiliations:** 1 Department of Pulmonary and Critical Care Medicine, The Fourth Hospital of China Medical University, Shenyang, 110032 People’s Republic of China; 2 Liaoning University of Traditional Chinese Medicine, Shenyang, 110167 People’s Republic of China; 3 Department of Pulmonary and Critical Care Medicine, Shengjing Hospital of China Medical University, Shenyang, 110004 People’s Republic of China; 4 Department of Pharmaceutical Toxicology, School of Pharmaceutical Sciences, China Medical University, Shenyang, 110001 People’s Republic of China

**Keywords:** Respiratory tract diseases, Medical research

## Abstract

Although diaphragmatic dysfunction is an important indicator of severity of illness and poor prognosis in ICU patients, there is no convenient and practical method to monitor diaphragmatic function. This study was designed to analyze diaphragmatic dynamic dysfunction by bedside ultrasound in septic patients and provide quantitative evidence to assess diaphragm function systematically. This prospective observational study was conducted from October 2019 to January 2021 in the Department of Respiratory and Critical Care Medicine. 74 patients suffered from sepsis were recruited and divided into two groups, sepsis group 1 (2 ≤ SOFA ≤ 5, n = 41) and sepsis group 2 (SOFA > 5, n = 33). 107 healthy volunteers were randomly recruited as the control group. In all participants, the diaphragmatic thickness and excursion were measured directly and the dynamic parameters including thickening fraction (TF), E_QB_/E_DB_, Contractile velocity, and area under diaphragmatic movement curve (AUDMC) were calculated by bedside ultrasound during quiet breathing (QB) and deep breathing (DB). Each parameter among three groups was analyzed separately by covariance analysis, which was adjusted by age, sex, body mass index, MAP, hypertension, and diabetes. First, contractile dysfunction occurred before diaphragmatic atrophy both in sepsis group 1 and sepsis group 2. Second, compared with the control group, the dynamic parameters showed significant decrease in sepsis group 1 and more obvious change in sepsis group 2, including TF, E_QB_/E_DB_. Third, the maximum contractile velocity decreased in sepsis group 1, reflecting the damage of intrinsic contraction efficiency accurately. Finally, per breathing AUDMC in two septic groups were lower than those in control group. However, per minute AUDMC was compensated by increasing respiratory rate in sepsis group 1, whereas it failed to be compensated which indicated gradual failure of diaphragm in sepsis group 2. Diaphragmatic ultrasound can be used to quantitatively evaluate the severity of sepsis patients whose contractile dysfunction occurred before diaphragmatic atrophy. As dynamic parameters, TF and E_QB_/E_DB_ are early indicator associated with diaphragmatic injury. Furthermore, maximum contractile velocity can reflect intrinsic contraction efficiency accurately. AUDMC can evaluate diaphragmatic breathing effort and endurance to overcome resistance loads effectively.

## Introduction

The diaphragm is the most important respiratory pump muscle, accounting for 60–80% of the respiratory dynamic^1^, and its dysfunction can cause respiratory failure directly. The application of mechanical ventilation and sepsis are two important risk factors for development of diaphragmatic injury in critical patients^2^. More than 50% of critical patients have severe diaphragmatic dysfunction, which is closely related to poor clinical prognosis^3–5^. Therefore, the diaphragm protection was proposed as important as the lung protection in the latest mechanical ventilation strategies^6^.

Unfortunately, there is no routine examination currently for monitoring the diaphragm in clinical setting. Although transdiaphragmatic pressure was an accurate measure of diaphragmatic function, it was mainly used in institutions because of its invasiveness^7–9^. In the last decade, bedside ultrasound has made a breakthrough in ventilator-induced diaphragmatic dysfunction (VIDD) and predicting weaning outcomes in mechanical ventilated patients^10–13^. However, studies on diaphragmatic ultrasound had rarely been reported in septic patients. Limited studies showed that bedside ultrasound was able to distinguish of diaphragmatic dysfunction from ventilated patients with sepsis to those without sepsis ^14–16^. Our previous study had shown that the diaphragmatic excursion and TF by bedside ultrasound had significantly decreased in septic patients^17^. This study was designed to analyze severity of diaphragmatic dynamic dysfunction by bedside ultrasound in septic patients and provide quantitative evidences to evaluate diaphragm function systematically.

## Methods

### Subjects and study design

This prospective observational study was conducted in the Department of Respiratory and Critical Care Medicine, and was approved (Approval number: 2019PS624K) by the Ethics Committee of Shengjing Hospital affiliated to China Medical University. The study was performed in accordance with the Declaration of Helsinki. Informed consent was signed by patients or their family members.

All the consecutively hospitalized septic patients were screened and enrolled into the study from October 2019 to January 2021 if they met the following criteria: (1) septic patients diagnosed according to the 2016 Sepsis and Septic Shock Third Edition International Consensus Definition with an acute change of SOFA score ≥ 2 on the basis of infection^18^, (2) at least 18 years old, (3) within the 24 h period after diagnosis for sepsis, and (4) without using mechanical ventilation, and (5) without using steroids, analgesics, and sedatives. We excluded septic patients complicated with mediastinal disease, neuromuscular disease, chronic obstructive pulmonary disease, hyperthyroidism or hypothyroidism, recent cardiac or thoracic surgery, and hemodynamic instability. Healthy volunteers were recruited as the control group during this period.

### Research protocol and collection of clinical data

All patients admitted for infection were assessed for SOFA scores at admission and every 24 h after admission. Patients who met the above mentioned criteria were enrolled in the study. Although there is no consensus on the severity stratification based on SOFA, studies had shown a significantly increased mortality in septic patients whose SOFA > 5^19,20^. Accordingly, the septic patients were stratified into two groups, sepsis group 1 (2 ≤ SOFA ≤ 5) and sepsis group 2 (SOFA > 5). All enrolled patients completed a bedside ultrasound assessment at a similar period (8 am ~ 5 pm) within 24 h of the diagnosis of sepsis and without using mechanical ventilation or before using mechanical ventilation.

Clinical data of participants were recorded including sex, age, body mass index (BMI), underlying disease (hypertension, diabetes), mean arterial pressure (MAP), and SOFA score.

### Diaphragmatic ultrasound measurement

The right hemidiaphragm of the participants was measured by GE Vivid IQ ultrasound color Doppler diagnostic instrument (Wuxi, China) in the supine position and original data and its dynamic parameters were recorded. The ultrasound measurements in the entire study were made by one trained ultrasound technician who received training in diaphragmatic ultrasound.

#### Diaphragmatic thickness and thickening fraction

**Figure 1:**
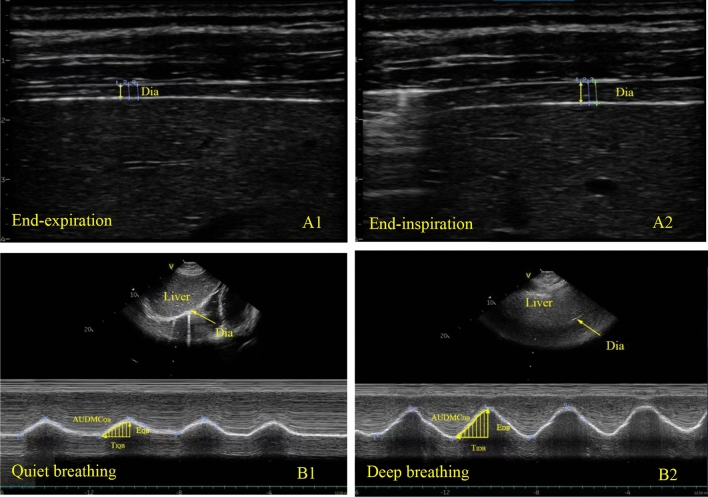
Diaphragmatic thickness measured by B mode ultrasound at end-expiration (**A1**) and end-maximum inspiration (**A2**). Diaphragmatic mobile curve in M mode of ultrasound was shown during quiet breathing (**B1**) and deep breathing (**B2**). Parameters were measured at three consecutive breathing cycles. EQB, excursion during quiet breathing; EDB, excursion during deep breathing; TI, inspiratory time; AUDMC, area under diaphragmatic movement curve in the inspiratory phase. Contractile velocity: diaphragmatic excursion/inspiratory time.

The linear array probe (9L-RS) with a frequency of 3.0–10 MHz was placed perpendicularly to chest wall close to the mid-axillary line which is between the 8th and 10th intercostal space. B-mode ultrasound was used to display the structure at the zone of apposition. The thickness of diaphragm was measured at the end of expiration and maximum inspiration for three times and the average values were recorded (Fig. 1A1,A2). Thickness of end-expiration was defined as diaphragmatic thickness^8^. Thickening fraction (TF) of diaphragm is calculated with the following formula: TF = [(Thickness at the end of maximum inspiration − Thickness at the end of expiration)/Thickness at end of expiration] × 100%.

#### Diaphragm movement curve

The convex array probe (M5SC-RS) with a frequency of 1.5–4.6 MHz was placed below the midclavicular line of the right costal margin in longitudinal scanning plane. The liver was used as an inspection window, and the probe was pointed toward the cephalic side. The ultrasound bundle was perpendicular to the posterior third of the right diaphragm. M-mode ultrasound was used to record the diaphragmatic movement curve during quiet breathing (QB) and deep breathing (DB). For ensuring all participants doing their best, we instructed them to breathe deeply from the functional residual position with maximum inspiratory effort repeatedly before diaphragmatic ultrasound was performed. Relevant parameters were measured in diaphragmatic movement curve (Fig. 1B1,B2): ① Diaphragmatic excursion (E) is vertical distance between the baseline and the peak of curve. ② E_QB_/E_DB_ is calculated by the following formula: diaphragmatic excursion during quiet breathing/excursion during deep breathing. ③ Inspiratory time is the horizontal distance from the starting point to the peak of the curve. ④ Contractile velocity is calculated by the following formula: diaphragmatic excursion/inspiratory time. ⑤ Per breathing AUDMC is the area under diaphragmatic movement curve in the inspiratory phase of a single breathing, whereas per minute AUDMC is that per breathing AUDMC times respiratory rate. Three successive respiratory cycle are measured, taking the average value of each parameter.

### Statistical analysis

Continuous variables with normal distribution were reported as mean±standard deviation (SD); non-normal variables were reported as median (interquartile range, IQR). Categorical variables were reported as frequency (percentage). The differences among characteristics were tested using analysis of variance, Kruskal–Walis H test, and chi-square test, respectively. Each diaphragmatic parameter among three groups was analyzed separately by covariance analysis. The *P* values for trend of diaphragmatic ultrasound parameters were calculated based on generalized linear models. Bonferroni was used for multiple comparisons among three groups. The above mentioned analyses were adjusted for sex, age, BMI, MAP, hypertension and diabetes, rather than using the raw data of diaphragmatic parameters directly. Spearman correlation analysis were performed to study the relationship between the AUDMC and TF. The *P *value was two tailed, and the significant difference was defined as *P *< 0.05.

For sample size, we calculated the statistical power for each parameter by PASS software according to the existing sample size, the mean, and the overall standard deviation of each group. The results show that the statistical power of each parameter was close to or more than 0.9 (Supplemental material). Considering the possible withdraw during the follow-up, we recruited the controls as many as possible during the participants enrolling in order that the number of controls was equal to or more than the number of the experimental group. The analysis was conducted using SAS version 9.4 (SAS Institute Inc., Cary, NC, USA). The figures were drawn using GraphPad 8.0. Normally distributed data is reported with a histogram, and non-normally distributed data is reported with a boxplot.

### Ethics approval and consent to participate

This study was approved by The Ethics Committee of Shengjing Hospital. The batch number is 2019PS624K. All patients or their family members provided signed informed consent.

## Results

### General characteristics

74 Septic patients were recruited in our study, of which 41 of 74 were sepsis group 1 and 33 of 74 were sepsis group 2, while 107 healthy volunteers were recruited as control group. All of septic patients were hospitalized with diagnosis of pulmonary infection, whose basic characteristics were shown in Table 1 together with control group. There was no significant difference in sex, age, and BMI among three groups. The mean arterial pressure (MAP) in sepsis group 2 was lower than that in control group and sepsis group 1 (*P* < 0.01, respectively). The prevalence of hypertension in septic patients was significantly higher than that in the control group, whereas there was no significant difference in prevalence of diabetes among the three groups.

**Table 1:** General characteristics in three groups.

Characteristics	Control (n = 107)	Sepsis1 (n = 41)	Sepsis2 (n = 33)	*P* value
Age [Mean (SD), year]	61.52 ± 11.48	60.98 ± 13.45	60.03 ± 14.95	0.86
Male (N, %)	54 (54.50)	24 (58.5)	18 (54.55)	0.67
BMI [Mean (SD), kg/m^2^]	23.37 ± 3.00	22.73 ± 2.97	22.77 ± 2.27	0.40
MAP [Mean (SD), mmHg]	85.75 ± 8.25	87.34 ± 8.58	78.45 ± 6.40	< 0.01
History of diabetes mellitus (N, %)	9 (8.41)	3 (7.32)	7 (21.21)	0.08
History of hypertension (N, %)	11 (10.28)	10 (24.39)	10 (30.30)	0.01

The data of categorical variables are presented as N (%), and the data of continuous variables are presented as mean ± SD.

### Diaphragmatic thickness

There was no significant difference in diaphragmatic thickness among sepsis group 1 [2.09(1.83–2.30) cm], sepsis group 2 [1.98(1.83–2.15) cm] and control group [1.96(1.79–2.11) cm], suggesting that there was no diaphragm atrophy in septic patients we studied (Table 2).

**Table 2:** Diaphragmatic thickness and excursion measured by ultrasound in three groups.

	Raw diaphragmatic parameter^a^			Adjusted model (least-square mean, 95% CI)^b^			*P* for trend^c^
	Control (n = 107)	Sepsis1 (n = 41)	Sepsis2 (n = 33)	Control (n = 107)	Sepsis1 (n = 41)	Sepsis2 (n = 33)	
Thickness(cm)	1.96(1.79–2.11)	2.09(1.83–2.30)	1.98(1.83–2.15)	1.96(1.79–2.11)	2.09(1.84–2.30)	1.98(1.83–2.14)	0.65
TF (%)	68(60–77)	46(39–55)	20(17–25)	68(60–77)	46(39–54)*	20(18–24)*^†^	< 0.0001
**Excursion (mm)**							
Quiet breathing	16.41 ± 3.92	15.31 ± 3.74	10.33 ± 2.49	16.19(15.09- 17.29)	14.84(13.46–16.21)	10.14(8.78–11.51)*^†^	< 0.0001
Deep breathing	42.26 ± 8.21	27.46 ± 6.03	16.27 ± 4.76	41.66(39.46–43.86)	27.14(24.41–29.88)*	16.01(13.30–18.72)*^†^	< 0.0001
E_QB_/E_DB_	0.38(0.34–0.46)	0.56(0.50–0.62)	0.67(0.58–0.70)	0.40(0.37–0.43)	0.56(0.52–0.60)*	0.66(0.62–0.69)*^†^	< 0.0001

The above analyses were adjusted for sex, age, BMI, MAP, hypertension and diabetes, rather than using the raw data of diaphragmatic parameters directly.

*P < 0.05 compared with control group; †*P* < 0.05 compared with sepsis group 1.

^a^All raw data was presented as median (IQR) or mean ± SD for continuous variables.

^b^The adjusted least-square mean and 95% CI were calculated by covariance analysis (CI = confidence interval).

^c^*P* for trend of diaphragmatic ultrasound parameters across sepsis severity were calculated based on generalized linear models.

### TF

**Figure 2:**
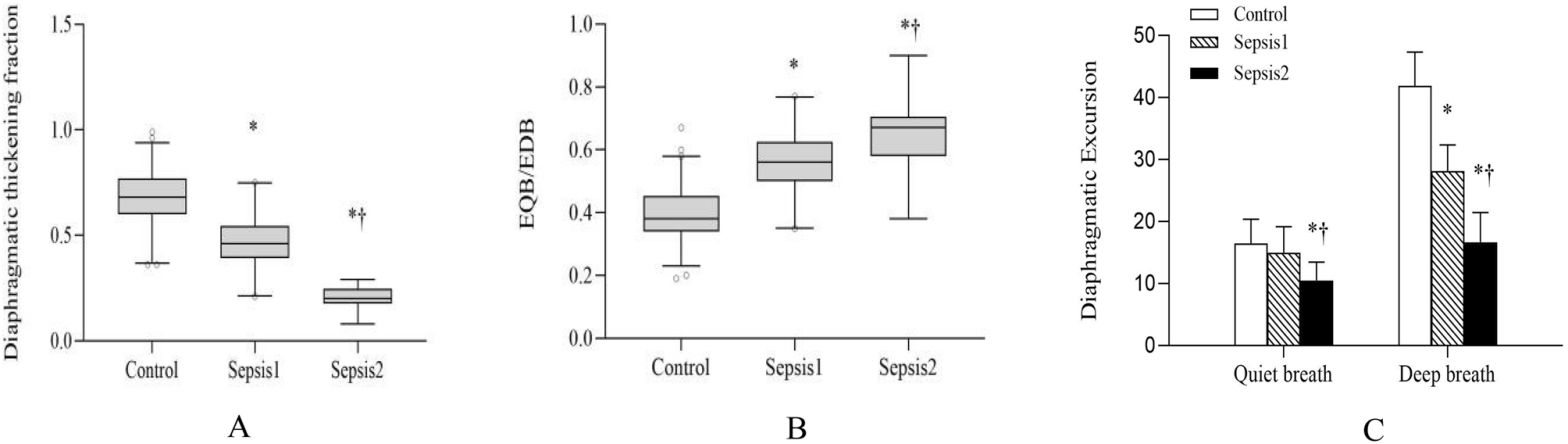
Diaphragmatic thickening fraction (**A**), EQB/EDB (**B**), excursion during quiet breathing and deep breathing (**C**). *P < 0.05 compared with control group; †*P* < 0.05 compared with sepsis group 1.

TF in sepsis group 1 [46(39–55) %] and 2 [20(17–25) %] were lower than that in control group [68(60–77) %] (*P* < 0.05), while TF in sepsis group 2 was lower than that in sepsis group 1 (*P* < 0.05) (Fig. 2A; Table 2).

### Diaphragm movement curve and function

#### Diaphragmatic excursion

The diaphragmatic excursion in sepsis group 2 [10.33 ± 2.49 mm] was lower than that in sepsis group 1 [15.31 ± 3.74 mm] and control group [16.41 ± 3.92 mm] during quiet breathing (*P* < 0.05), but there was no significant difference between sepsis group 1 and control group. However, during deep breathing, the diaphragmatic excursion were both lower in sepsis group 1 [27.46 ± 6.03 mm] and sepsis group 2 [16.27 ± 4.76 mm] than that in control group [42.26 ± 8.21 mm] (*P* < 0.05), and sepsis group 2 had a further downward trend than that in sepsis group 1(*P* for trend < 0.05) (Fig. 2C; Table 2).

#### E_QB_/E_DB_

The E_QB_/E_DB_ was both increased in sepsis group 1 [0.56(0.50–0.62)] and sepsis group 2 [0.67(0.58–0.70)] than that in control group [0.38(0.34–0.46)] (*P* < 0.05). Nevertheless, it was higher in sepsis group 2 than that in the sepsis group 1 (*P* < 0.05) (Fig. 2B; Table 2).

#### Inspiratory time

The inspiratory time was significantly shortened in sepsis group 1 and sepsis group 2 than that in control group no matter during quiet breathing or deep breathing (*P* < 0.05). Although there was no significant difference in inspiratory time between sepsis group1 and sepsis group 2 during quiet breathing, it was shortened significantly in sepsis group 2 during deep breathing (Table 3).

**Table 3:** Diaphragmatic contractile velocity and AUDMC measured by ultrasound in three groups.

	Raw diaphragmatic parameter^a^			Adjusted model (least-square mean, 95% CI)^b^			*P* for trend^c^
	Control (n = 107)	Sepsis1 (n = 41)	Sepsis2 (n = 33)	Control (n = 107)	Sepsis1 (n = 41)	Sepsis2 (n = 33)	
Inspiratory time (QB) (ms)	1401.00 (1230.00–1590.00)	990.00 (911.50–1145.00)	1001.00 (845.00–1060.00)	1341.87 (1275.56–1408.17)	991.18 (908.63–1073.73)*	943.54 (861.77–1025.31)*	< 0.0001
Inspiratory time (DB) (ms)	1710.00 (1412.00–1900.00)	1306.00 (1118.50–1557.50 )	1137.00 (995.00–1194.00)	1628.89 (1530.96–1726.82)	1324.82 (1202.90–1446.75)*	1045.94 (925.17–1166.70)*^†^	< 0.0001
Contractile velocity (QB) (mm/s)	1.13 (1.00–1.32)	1.50 (1.37–1.67)	1.03 (0.89–1.23)	1.22 (1.11–1.33)	1.50 (1.36–1.63)*	1.13 (0.99–1.26)^†^	0.21
Contractile velocity (DB) (mm/s)	2.42 (2.14–2.77)	2.00 (1.75–2.37)	1.32 (1.22–1.86)	2.65 (2.40–2.90)	2.08 (1.77–2.39)*	1.58 (1.27–1.88)*^†^	< 0.0001
AUDMC (QB) (cm-s)	7.57 (6.65–9.52 )	5.94 (4.01–6.72 )	2.77 (2.01–3.58)	11.93 (11.01–12.84)	8.07 (6.93–9.23)*	5.16 (4.02–6.30)*^†^	< 0.0001
AUDMC (DB) (cm-s)	32.99 (26.50–39.21)	17.80 (14.09–25.14)	6.13 (4.44–9.04)	35.57 (33.33–37.81)	19.29 (16.48–22.06)*	9.04 (6.29–11.81)*^†^	< 0.0001
AUDMC (per minute) (cm-s)	148.29 ± 42.56	134.79 ± 39.41	81.23 ± 34.06	215.46 (198.54–232.38)	199.25 (178.18–220.31)	144.75 (123.88–165.62)*^†^	< 0.0001

The above analyses were adjusted for sex, age, BMI, MAP, hypertension and diabetes, rather than using the raw data of diaphragmatic parameters directly.

*P < 0.05 compared with control group; †*P* < 0.05 compared with sepsis group 1.

^a^All raw data was presented as median (IQR) or mean ± SD for continuous variables.

^b^The adjusted least-square mean and 95% CI were calculated by covariance analysis (CI = confidence interval).

^c^*P* for trend of diaphragmatic ultrasound parameters across sepsis severity were calculated based on generalized linear models.

#### Contractile velocity

**Figure 3:**
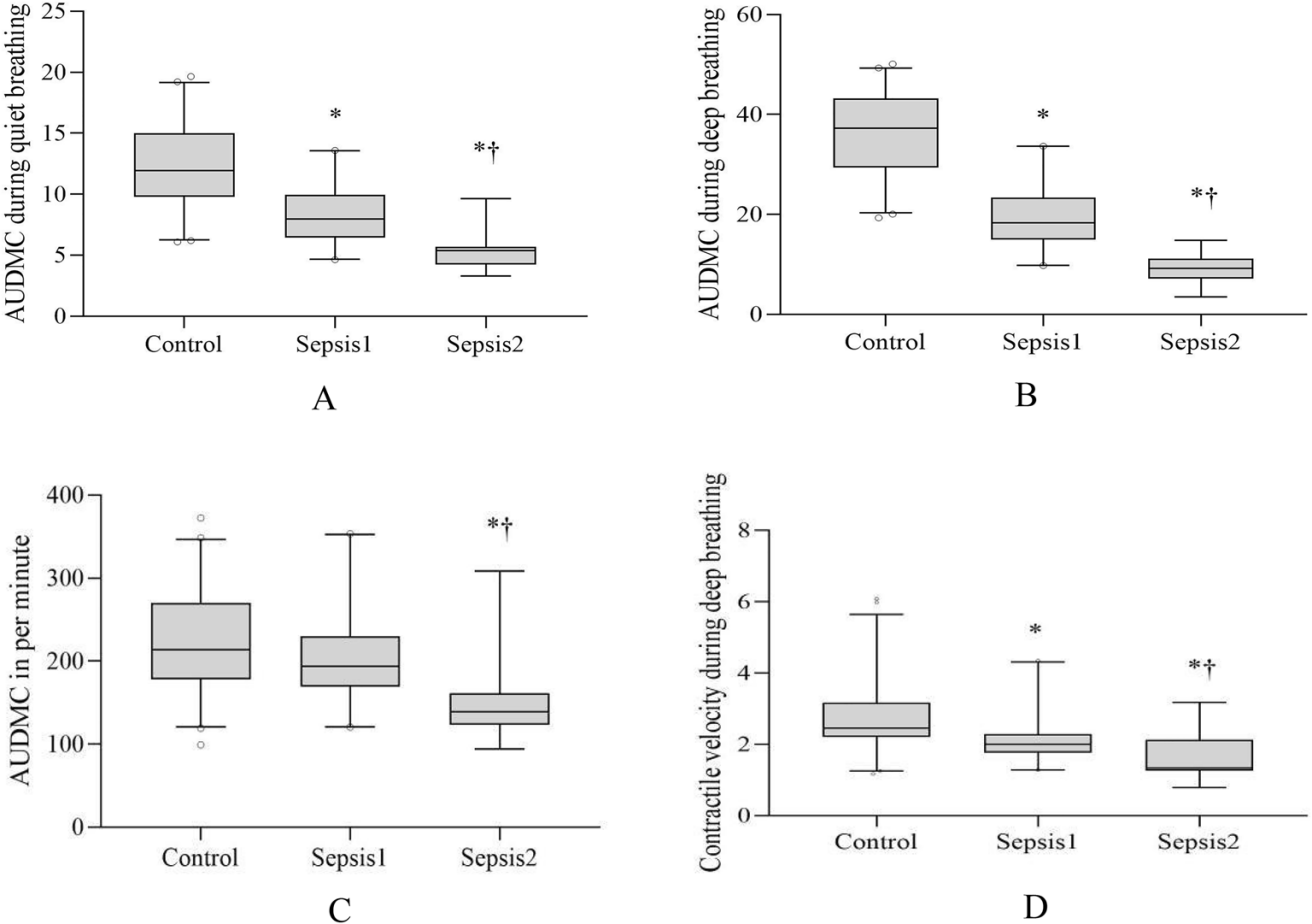
AUDMC during quiet breathing (**A**), AUDMC during deep breathing (**B**), AUDMC in per minute (**C**), Diaphragm contractile velocity (**D**). *P < 0.05 compared with control group; †*P* < 0.05 compared with sepsis group 1.

Maximum contractile velocity, contractile velocity during deep breathing, decreased in sepsis group 1 [2.00(1.75–2.37) mm/s] and sepsis group 2 [1.32(1.22- 1.86) mm/s] compared with the control group [2.42(2.14–2.77) mm/s] (*P* < 0.05), which showed a linear downward trend among three groups (Fig. 3D). During quiet breathing, the diaphragmatic contractile velocity was significantly higher in the sepsis group 1 [1.50(1.37–1.67) mm/s] than that in control group [1.13(1.00–1.32) mm/s] and sepsis group 2 [1.03(0.89–1.23) mm/s] (*P* < 0.05), though there was no statistical difference between the two groups (*P* > 0.05) (Table 3).

#### AUDMC in the inspiratory phase

We measured the area under diaphragmatic movement curve (AUDMC) in the inspiratory phase of breathing. Per breathing AUDMC in two septic groups was lower than that in control group both during quiet breathing and deep breathing (*P* < 0.05)(Fig. 3A,B), and showed a linear downward trend among three groups (*P* for trend < 0.05). Per minute AUDMC in sepsis group 2 were significantly decreased compared with sepsis group 1 and control group (*P* < 0.05, respectively) (Fig. 3C). However, there was no significant difference between sepsis group 1 and control group in the per minute AUDMC (*P* > 0.05) (Table 3).

We calculated the correlations between the AUDMC and TF. The results suggested that the positive Spearman correlation coefficients were significant between the AUDMC and TF [during deep breath: *r* = 0.61 (95% CI 0.50–0.69), *P* < 0.05, quite breathing:* r* = 0.59 (95% CI 0.48–0.68), *P* < 0.05].

## Discussion

Our prospective observational study provided evidence to evaluate sepsis-induced diaphragm dysfunction (SIDD) by bedside ultrasound systematically. The parameters of diaphragmatic mobility were used to quantify during both deep breathing and quiet breathing among sepsis group 1 (2 ≤ SOFA ≤ 5), sepsis group 2 (SOFA > 5) and control group. The major findings were as follows: (1) The contractile dysfunction occurred before diaphragmatic atrophy in septic patients. (2) The severity of diaphragm dysfunction can be quantitatively analyzed by ultrasound parameters in septic patients. The dynamic parameters including TF, E_QB_/E_DB_, compared with deep breathing and quiet breathing, reflected dysfunction earlier. (3) The maximum contractile velocity decreased significantly in sepsis group 1, reflecting the damage of intrinsic contraction efficiency accurately. (4) AUDMC (either per breathing or per minute), the integral of the diaphragmatic excursion during contraction over time, decreased in septic patients in our study, which reflected a reduced breathing effort of diaphragm to overcome resistance loads effectively.

In fact, diaphragmatic dysfunction was both a marker of severity of disease and a predictor of poor outcomes in ICU patients^7^. The previous studies of diaphragmatic ultrasound mostly focused on predicting the probability weaning from mechanically ventilated in ICU, which relating parameters included thickness, TF and Excursion^12,13,21^. Of these, TF is much more accuracy than excursion^22^. In our study, abnormal TF and E_QB_/E_DB_ achieved better assessment of diaphragm dysfunction in septic patients early. Because they reflected the relationship between contractile function in quiet breathing and maximum contractile reserve.

The diaphragm function depends not only on the strength of contractile force but also on the velocity of diaphragm contraction. The variation in the thoracic pressure generated by maximum inspiratory effort is mainly determined by the intrinsic contractile properties of the diaphragm and is not affected by pulmonary compliance or structural changes in the chest wall, which can better reflect the diaphragmatic contraction efficiency accurately^23,24^. In our study, the maximum contractile velocity, reflecting the fastest speed of diaphragm contraction per unit time, decreased significantly in septic groups comparing with that in control group, and decreased more significantly in sepsis group 2 than that in sepsis group 1.

Actually, the same excursion could represent different breathing efforts because inspiratory time and respiratory rate are not taken into account^25^. Area under diaphragmatic movement curve (AUDMC) in the inspiratory phase is the integral of the diaphragmatic excursion during contraction over time (either per breathing or per minute). During inspiratory phase, the shortening of the diaphragm fibers produced a piston-like downward action, which the radius of diaphragmatic dome-like structure changes from time to time^7,10,26^. Our study found a positive correlation between the TF and AUDMC. Because TF has been proved to reflect the breathing efforts by the research of Goligher et al^12^, AUDMC can also reflect diaphragmatic effort. Whether AUDMC covering inspiratory process can represent respiratory effort accurately needs to be confirmed by further studies in future. Furthermore, per minute AUDMC was calculated to better predict the pump function and endurance of diaphragm. In our study, per breathing AUDMC in sepsis group 1 was lower than that in control group, but per minute AUDMC was compensated by increasing respiratory rate. However, per minute AUDMC in sepsis group 2 failed to be compensated, which indicated gradual failure of diaphragm.

Sepsis is a series of clinical syndromes caused by infection, resulting in multiple organ dysfunction, including diaphragmatic dysfunction. Several cellular and molecular mechanisms have been involved in SIDD, including Oxidative stress and the activation of multiple proteolytic pathways^2^. Sepsis-induced diaphragmatic dysfunction includes the two aspects of decreased diaphragm contractility and diaphragm atrophy^2^. We found that contractile dysfunction was earlier than diaphragmatic atrophy in septic patients of our study, which had confirmed by basic and clinical studies^16,27^. With the aggravation of infection severity, we found a further decrease in the diaphragmatic contractile function by ultrasound, including parameters of TF, E_QB_/E_DB,_ velocity in deep breathing and AUDMC. Therefore, the technique of diaphragm ultrasound can be used to evaluate both diaphragmatic contraction function and structure change in septic patients.

Although there is no international standard for diaphragmatic ultrasound techniques compared to cardiac ultrasound, it has been increasingly studied in recent years because it is noninvasive and has no potential radioactive damages like CT and X-ray^5,7,21^. In order to rule out the influence of clinically relevant confounding factors, the covariance analysis was performed for each parameter separately, which adjusted by age, sex, body mass index, MAP, hypertension, and diabetes in our study. Among them, the dynamic evaluation parameters including TF and E_QB_/E_DB_ were compared quiet breathing with maximum inspiration in patients themselves, which reduced the errors caused by different individuals and different instruments.

All septic patients were grouped based on SOFA scores because it is not only as diagnostic criteria, but also as more important evidence to help clinicians identify the severity of infection early and initiate timely treatment^28^. By comparing the more severe sepsis group 2 with that of the sepsis group 1, we found that some parameters by diaphragmatic ultrasound can be used to assess diaphragmatic injury earlier. So this study provides a basis for routine clinical evaluation of diaphragmatic function in the future. For ruling out the additional influence on diaphragmatic function, all the patients were performed diaphragmatic ultrasound before mechanical ventilation. Of course, these parameters also need to be studied in other septic patients and clinical illness.

## Limitation

The right hemidiaphragm of all participants was measured in supine position because the intra-abdominal pressure in supine is lower than that of other position and the right hemidiaphragm has better repeatability than the left one^29^. Unfortunately, there is no universal approach for position of diaphragm ultrasound, and we need to determine the best approach when we encounter more specific clinical problems or setting. Furthermore, since our study is a single-center study, further studies with multiple centers and participants are needed to obtain more reliable results.

## Conclusions

Diaphragmatic ultrasound can be used to quantitatively evaluate the severity of septic patients whose contractile dysfunction occurred before diaphragmatic atrophy. As dynamic parameters, TF and E_QB_/E_DB_ are early indicator associated with diaphragmatic injury. Furthermore, maximum contractile velocity can reflect intrinsic contraction efficiency accurately. Per breathing AUDMC and per minute AUDMC can evaluate diaphragmatic breathing effort and endurance to overcome resistance loads effectively because they take inspiratory time into account.

## Supplementary Information

Supplementary Information.

## Data Availability

The data that support the findings of this study are available from the corresponding author upon reasonable request.
