# Supplementary material for: Quantification of diaphragmatic dynamic dysfunction in septic patients by bedside ultrasound
Source: Sci Rep. 2022 Oct 15;12:17336. doi: 10.1038/s41598-022-21702-6 (PMC9569367; doi:10.1038/s41598-022-21702-6)
Supplement: Supplementary file 1 — Supplementary Information. [file 41598_2022_21702_MOESM1_ESM.pdf]

Supplement

Quantification of Diaphragmatic Dynamic Dysfunction in Septic Patients by Bedside Ultrasound

Yunqiu Chen1 # , Yujia Liu2 # , Mingxin Han3, Shuai Zhao3, Ya Tan3, Liying Hao4, Wenjuan Liu3, Wenyan Zhang3, Wei Song3, Mengmeng Pan3, Guangyu Jiao*3

1. Department of Pulmonary and Critical Care Medicine, The Fourth Hospital of China Medical University, Shenyang, People's Republic of China,110032
2. Liaoning University of Traditional Chinese Medicine,Shenyang, People's Republic of China, 110167
3. Department of Pulmonary and Critical Care Medicine, Shengjing Hospital of China Medical University, Shenyang, People's Republic of China,110004
4. Department of Pharmaceutical Toxicology, School of Pharmaceutical Sciences, China Medical University, Shenyang, People's Republic of China, 110001

*Corresponding author：Guangyu Jiao, E-mail:jiao_gy@163.com

# Co-first authors contribute equally

About sample size:

We calculated the statistical power for each parameter by PASS software according to the existing sample size, the mean, and the overall standard deviation of each group. The results show that the statistical power of each parameter was close to or more than 0.9 (Table S1).

Table S1 Statistical power of each parameter

	Power	Total Sample Size	Base Subjects Per Group	Standard Deviation of Group Means(σm)	Standard Deviation (σ)	Effect Size	Alpha
TF	1.0000	181	33	18.47	26.05	0.709	0.05
Excursion(QB)	1.0000	181	33	2.26	4.77	0.474	0.05
Excursion(DB)	1.0000	181	33	10.18	9.50	1.072	0.05
E_{QB}/E_{DB}	1.0000	181	33	0.11	0.13	0.808	0.05
Inspiratory time(QB)	1.0000	181	33	183.48	286.42	0.641	0.05
Inspiratory time(DB)	1.0000	181	33	228.52	423.03	0.540	0.05
Contractile velocity(QB)	0.9201	181	33	0.13	0.47	0.276	0.05
Contractile velocity(DB)	0.9982	181	33	0.42	1.08	0.391	0.05
AUDMC(QB)	1.0000	181	33	2.70	3.98	0.679	0.05
AUDMC(DB)	1.0000	181	33	10.76	9.68	1.112	0.05
AUDMC(per minute)	0.9940	181	33	26.40	73.09	0.361	0.05
Thickness	0.8958	181	33	0.05	0.2	0.265	0.05

Note: QB means quite breathing and DB means deep breathing.

The detailed calculation is as follows:

TF

One-Way Analysis of Variance F-Tests

Numeric Results

Number of Groups: 3

Power	Total Sample Size N	Group Sample Size Set Ni Set	Base Subjects Per Group Ni	Group Means Set μi	SD of Group Means σm	Std Dev σ	Effect Size σm/σ	Alpha
1.0000	181	Ni(1)	33	μi(1)	18.47	26.05	0.709	0.050

Excursion (quiet breathing)

One-Way Analysis of Variance F-Tests

Numeric Results

Number of Groups: 3

Power	Total Sample Size N	Group Sample Size Set Ni Set	Base Subjects Per Group Ni	Group Means Set μi	SD of Group Means σm	Std Dev σ	Effect Size σm/σ	Alpha
1.0000	181	Ni(1)	33	μi(1)	2.26	4.77	0.474	0.050

Excursion(deep breathing)

One-Way Analysis of Variance F-Tests

Numeric Results
Number of Groups: 3

Power	Total Sample Size N	Group Sample Size Set Ni Set	Base Subjects Per Group Ni	Group Means Set μi	SD of Group Means σm	Std Dev σ	Effect Size σm/σ	Alpha
1.0000	181	Ni(1)	33	μi(1)	10.18	9.50	1.072	0.050

E_{QB}/E_{DB}

One-Way Analysis of Variance F-Tests

Numeric Results
Number of Groups: 3

Power	Total Sample Size N	Group Sample Size Set Ni Set	Base Subjects Per Group Ni	Group Means Set μi	SD of Group Means σm	Std Dev σ	Effect Size σm/σ	Alpha
1.0000	181	Ni(1)	33	μi(1)	0.11	0.13	0.808	0.050

Inspiratory time(ms)--

Quiet breathing

One-Way Analysis of Variance F-Tests

Numeric Results
Number of Groups: 3

Power	Total Sample Size N	Group Sample Size Set Ni Set	Base Subjects Per Group Ni	Group Means Set μi	SD of Group Means σm	Std Dev σ	Effect Size σm/σ	Alpha
1.0000	181	Ni(1)	33	μi(1)	183.48	286.42	0.641	0.050

Inspiratory time(ms)--

Deep breathing

One-Way Analysis of Variance F-Tests

Numeric Results
Number of Groups: 3

Power	Total Sample Size N	Group Sample Size Set Ni Set	Base Subjects Per Group Ni	Group Means Set μi	SD of Group Means σm	Std Dev σ	Effect Size σm/σ	Alpha
1.0000	181	Ni(1)	33	μi(1)	228.52	423.03	0.540	0.050

Contractile velocity(mm/s)-- Quiet breathing

One-Way Analysis of Variance F-Tests

Numeric Results
Number of Groups: 3

Power	Total Sample Size N	Group Sample Size Set Ni Set	Base Subjects Per Group Ni	Group Means Set μi	SD of Group Means σm	Std Dev σ	Effect Size σm/σ	Alpha
0.9201	181	Ni(1)	33	μi(1)	0.13	0.47	0.276	0.050

Contractile velocity(mm/s)-- Deep breathing

One-Way Analysis of Variance F-Tests

Numeric Results
Number of Groups: 3

Power	Total Sample Size N	Group Sample Size Set Ni Set	Base Subjects Per Group Ni	Group Means Set μi	SD of Group Means σm	Std Dev σ	Effect Size σm/σ	Alpha
0.9982	181	Ni(1)	33	μi(1)	0.42	1.08	0.391	0.050

AUDMC(cm-s)-- Quiet breathing

One-Way Analysis of Variance F-Tests

Numeric Results
Number of Groups: 3

Power	Total Sample Size N	Group Sample Size Set Ni Set	Base Subjects Per Group Ni	Group Means Set μi	SD of Group Means σm	Std Dev σ	Effect Size σm/σ	Alpha
1.0000	181	Ni(1)	33	μi(1)	2.70	3.98	0.679	0.050

AUDMC(cm-s)-- Deep breathing

One-Way Analysis of Variance F-Tests

Numeric Results
Number of Groups: 3

Power	Total Sample Size N	Group Sample Size Set Ni Set	Base Subjects Per Group Ni	Group Means Set μi	SD of Group Means σm	Std Dev σ	Effect Size σm/σ	Alpha
1.0000	181	Ni(1)	33	μi(1)	10.76	9.68	1.112	0.050

AUDMC(cm-s)-- per minute

One-Way Analysis of Variance F-Tests

Numeric Results

Number of Groups: 3

Power	Total Sample Size N	Group Sample Size Set Ni Set	Base Subjects Per Group Ni	Group Means Set μi	SD of Group Means σm	Std Dev σ	Effect Size σm/σ	Alpha
0.9940	181	Ni(1)	33	μi(1)	26.40	73.09	0.361	0.050

Thickness

One-Way Analysis of Variance F-Tests

Numeric Results

Number of Groups: 3

Power	Total Sample Size N	Group Sample Size Set Ni Set	Base Subjects Per Group Ni	Group Means Set μi	SD of Group Means σm	Std Dev σ	Effect Size σm/σ	Alpha
1.0000	181	Ni(1)	33	μi(1)	0.05	0.10	0.530	0.050
0.8958	181	Ni(1)	33	μi(1)	0.05	0.20	0.265	0.050

References

1.Desu, M. M. and Raghavarao, D. 1990. Sample Size Methodology. Academic Press. New York.

2.Fleiss, Joseph L. 1986. The Design and Analysis of Clinical Experiments. John Wiley & Sons. New York.

3.Kirk, Roger E. 1982. Experimental Design: Procedures for the Behavioral Sciences. Brooks/Cole. Pacific Grove, California.
